# Smartphone Application With Health Coaching Facilitates Multi‐Symptom Improvement in IBS Patients: A Pilot Feasibility Trial

**DOI:** 10.1111/nmo.70179

**Published:** 2025-10-07

**Authors:** Max Eisele, Munazza Yousuf, Natasha Haskey, Adrijana D'Silva, Yasmin Nasser, Laura Franco, Maitreyi Raman

**Affiliations:** ^1^ Cumming School of Medicine University of Calgary Calgary Alberta Canada; ^2^ Department of Biology University of British Columbia—Okanagan Kelowna British Columbia Canada; ^3^ LyfeMD Platform Calgary Alberta Canada

**Keywords:** diet, health coaching, irritable bowel syndrome (IBS), lifestyle, mobile health (mHealth), physical activity

## Abstract

**Background:**

Irritable bowel syndrome (IBS), a disorder of the gut‐brain interaction, is associated with significant symptom burden and impaired psychosocial functioning. Evidence‐based behavioral therapies are effective, but often underutilized due to accessibility barriers. Mobile health is an emerging field with the potential to bridge the gap between the needs of individuals with IBS and the limitations of the healthcare system. This study evaluated the feasibility and effectiveness of the LyfeMD app plus health coaching (HC) in improving IBS symptom severity and psychosocial wellbeing.

**Methods:**

This 12‐week interventional pilot study evaluated the effectiveness of a mobile application combined with HC in adults diagnosed with IBS. Participants were assessed at baseline, 6 weeks, and 12 weeks using validated surveys to assess symptom severity, psychosocial wellbeing, diet, physical activity, and sleep. A Fitbit was also used to track physical activity and sleep.

**Results:**

Thirty‐nine participants completed the 12‐week intervention. IBS symptom severity improved significantly (*p* < 0.001) over the 12‐week period, with 63.2% of the participants having a clinically meaningful improvement in their symptoms. In addition to symptom severity, participants improved in all measured psychosocial domains and their subjective sleep quality at 12 weeks.

**Conclusion:**

In summary, the LyfeMD platform, in combination with HC, shows potential in improving IBS symptom severity, psychosocial well‐being, and sleep quality in individuals diagnosed with IBS. These findings highlight the potential of mobile health as a complement to traditional medical care. Further research, including randomized controlled trials with extended follow‐up, is needed to confirm findings and the sustainability of these outcomes.

## Graphical abstract

Benefits of a smartphone application and health coaching in IBS patients.

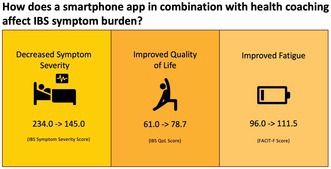

**Summary**

- Mobile health (mHealth) platforms such as LyfeMD have the potential to improve symptom severity, psychosocial wellbeing, and sleep in patients with irritable bowel syndrome, offering an effective, accessible, and low‐cost method to complement traditional medical care.

## 1 Introduction

Irritable bowel syndrome (IBS) is a chronic functional disorder of the gastrointestinal tract affecting 11% of the population globally, with rates in Canada estimated to be as high as 13.5% [1]. The etiology of IBS is not fully understood but is hypothesized to include a multifactorial bi‐directional dysregulation of the gut‐brain axis, resulting in visceral hypersensitivity, altered gastric and intestinal motility, gut microbial dysbiosis, and central nervous system connectivity [2, 3]. Due to the heterogeneity of IBS, it is subcategorized into individuals with diarrhea‐predominant (IBS‐D), constipation‐predominant (IBS‐C), or mixed pattern symptoms (IBS‐M). In addition to the symptom burden—including but not limited to increased stool frequency, abdominal pain, and bloating, individuals with IBS suffer from threefold higher rates of comorbid anxiety and depression compared to healthy adults [4]. Patients with IBS with anxiety or depression disproportionately suffer from worse gastrointestinal symptoms, quality of life (QoL), and fatigue [5], highlighting the need for a holistic management strategy to address these comorbidities in the treatment of IBS. Medical management of IBS tends to focus largely on symptom management, rather than targeting the etiopathogenetic factors, leaving many patients dissatisfied with their medical care [6]. Accordingly, IBS guidelines consistently recommend dietary therapy and behavioral modifications, alongside pharmacological management, as first‐line treatment strategies aimed at enhancing brain‐gut connectivity and psychological risk factors [7, 8, 9].

The primary dietary approaches studied for IBS management include the NICE guidelines, low fermentable oligosaccharides, disaccharides, monosaccharides, and polyols (FODMAP) diet, and increased intake of soluble fibre [7, 8, 9, 10]. The NICE guidelines focus on consuming regular meals, adequate fluid intake, and limiting insoluble fiber, caffeine, alcohol, and carbonated drinks. Additionally, should symptoms persist despite these changes, the NICE guidelines recommend the low FODMAPs diet [7]. In a network meta‐analysis, a low FODMAPs diet has been shown to be superior to all other diet interventions for improving abdominal pain and bloating [11]. Soluble fiber supplementation was found to improve symptom burden, particularly in IBS‐C patients [12].

The most studied and effective behavioral modifications are psychological therapies, specifically cognitive behavioral therapy (CBT) [9, 13] and gut‐directed hypnosis [14, 15]. Gut‐directed hypnotherapy modulates postprandial gastro‐colic reflex activity, alters colonic motility, reduces visceral hypersensitivity, and normalizes gut‐brain pain processing signals, which has been confirmed by functional brain imaging [14]. Additionally, some early evidence suggests that CBT may improve autonomic dysfunction, as measured by heart rate variability [16]. A recent systematic review sheds light on the effectiveness of CBT in improving high comorbid anxiety and depression rates, with the majority of studies showing significant improvement [17]. Finally, there is emerging literature for the efficacy of Acceptance and Commitment therapy (ACT) in IBS, a third‐wave behavioral therapy whose principles are often incorporated in breathing and mindfulness practices [18, 19].

Physical activity has been shown to improve clinical outcomes in IBS. It has been demonstrated to modulate gut transit time, optimize autonomic and immune system function—by decreasing systemic inflammation and enhancing mucosal immunity through the increased synthesis of IgA [20]. Individuals with IBS are recommended to at least attain 150 min of moderate to vigorous physical activity (MVPA) per week [20]. Little is known about the actual physical activity patterns of individuals with IBS, and although preliminary findings suggest that increasing physical activity (PA) levels may improve symptom burden, the studies are generally of poor quality [21].

Without additional support, the recommended behavioral changes and dietary therapies discussed in the clinic setting can be challenging to implement and maintain, which is reflected in persistent poor diet quality and low levels of physical activity among patients living with IBS [8, 22]. The heterogeneous nature of IBS, coupled with high rates of anxiety and depression, further underscores the need for personalized, multipronged interventions to help patients meet treatment guidelines. Health coaching (HC) is uniquely positioned to address these challenges by enhancing an individual's self‐efficacy to achieve desired behavior changes, often incorporating techniques such as motivational interviewing derived from CBT [23]. However, interventions like CBT and gut‐directed hypnotherapy are limited by high delivery costs [14] and limited accessibility [24].

Mobile health (mHealth) tools—including apps, phone calls, text messaging, and websites—offer a promising alternative to traditional methods of delivering behavior‐based interventions. These tools can enhance accessibility by lowering the barrier to entry, enhancing adherence, reducing strain on healthcare resources, and by offering a more cost‐effective solution [25]. The LyfeMD platform, developed by researchers at the University of Calgary specifically for individuals with IBS, delivers personalized behavior programs tailored to individual needs. It offers dietary and nutrition guidance, PA routines, yoga, breathing, and mindfulness (YBM) practices, gut‐directed hypnotherapy, and self‐guided CBT. Additionally, LyfeMD includes an integrated HC component that allows individuals to communicate with health coaches via scheduled calls or app‐based messaging. Despite emerging evidence supporting the effectiveness of mHealth tools in IBS management, there remains an overall lack of comprehensive research that simultaneously addresses all three core domains—diet, PA, and psychosocial well‐being [26].

The primary objective of this pilot study was to evaluate the effectiveness of the LyfeMD platform combined with HC in reducing the IBS symptom burden. Secondary objectives included assessing the effectiveness of the intervention on psychological well‐being, sleep quality, fatigue, and QoL in this population. We also examined the feasibility of recruitment, intervention delivery, and assessment completion. We hypothesize that a multimodal mHealth approach, such as the LyfeMD platform plus HC, will be both effective and feasible, leading to improvement in IBS symptom severity and psychosocial outcomes.

## 2 Methods and Materials

### 2.1 Study Design

This was a 12‐week, single‐center, pre‐post pilot study designed to evaluate the feasibility and effectiveness of the LyfeMD platform—a lifestyle mobile application combined with the support of a Health Coach, in adults diagnosed with IBS. Ethics approval was obtained by the University of Calgary Conjoint Health Research Ethics Board (REB22‐0482) and informed consent was provided prior to participation. Eligible participants were contacted and recruited between March 2023 and June 2024. Data collection and study management were conducted using the Research Electronic Data Capture (REDCap) [27] tool. The CONSORT Extension to Pilot and Feasibility Trials checklist was completed and can be found in the Supporting Information S2.

### 2.2 Participants

Adults aged 18 years or older with a diagnosis of IBS based on Rome IV criteria and having access to a smartphone were eligible to participate. Patients were recruited from previous research participations at the University of Calgary or during routine appointments at the gastroenterology clinics at the Foothills Medical Centre in Calgary, Alberta, Canada. Patients were excluded if they had a diagnosis of Inflammatory Bowel Disease (IBD), an inability to access the application due to language or technology barriers, recent major changes in their diet or physical activity, and physical limitations that would prevent participation in physical activity or yoga.

### 2.3 Intervention

The LyfeMD platform, originally developed at the University of Calgary, Alberta, Canada in May 2021, consists of a mobile app with supplementary video‐based modules. It was designed to provide patients with evidence‐based lifestyle therapies tailored to patients living with IBS. This comprehensive digital platform features programs focused on diet and nutrition, YBM, self‐administered Cognitive Behavioral Therapy (CBT), and PA. Upon logging into the app, participants completed a series of surveys, and the app generated scores assessing IBS severity, risk for anxiety and depression, levels of perceived stress, PA, and sleep quality. The generated scores helped participants and health coaches set personalized targets for the program selection (e.g., diet, yoga, PA, etc.), as well as frequency and duration of the engagement. Progress toward these targets was tracked weekly within the app, with reassessments conducted at weeks 6 and 12 to update scores, evaluate goal progress, and adjust goals as needed.

Participants completed baseline assessments before their first consultation with the HC. During this consultation, conducted via phone or video call, the HC provided an orientation to the app, addressed technological questions, and engaged in a general discussion about the participant's IBS experience—including symptoms, prior management strategies, and existing barriers. Based on this conversation and the survey scores described previously, the HC and participant collaboratively selected an initial program focus, typically focusing on dietary modifications, YBM practices, or gut‐directed hypnotherapy.

If dietary modification was chosen as the initial focus, the HC recommended a low FODMAP diet to participants with an IBS Symptoms Severity Scale (IBS‐SSS) score exceeding 200, while those with a score below 200 were advised to follow a general IBS elimination diet according to the NICE guidelines [7]. Dietary recommendations were personalized to align with patient preferences to maximize adherence. In circumstances whereby patients' survey scores indicated at least moderate risk for either anxiety or depression, recommendations for YBM or gut‐directed hypnotherapy via the LyfeMD modules were made as a first‐line program. All participants were offered to use the LyfeMD modules (except the first six participants at their time of participation; the LyfeME modules were not finalized), which consisted of videos with YBM components crafted based on findings from a published study in IBS populations [28], and gut‐directed hypnotherapy developed specifically for this study. Finally, the PA program encouraged adherence to public health guidelines, recommending at least 150 min of weekly moderate‐intensity exercise and strength training on two or more days, and guided by videos [29].

Follow‐up consultations between the HC and participants were scheduled for 2 weeks after the initial visit, with additional check‐ins at 6 and 12 weeks. Participants could contact the HC via email or text for further guidance outside of these scheduled times as needed. These follow‐ups supported progress tracking, problem‐solving around reported barriers, and the adjustment or introduction of new goals and app‐based programs as needed.

### 2.4 Outcome Measures

#### 2.4.1 Primary Outcomes

##### 2.4.1.1 Assessment of Disease Severity

IBS disease severity was assessed at baseline, week 6, and week 12 using the IBS‐SSS [30]. An improvement of ≥ 50 points on the IBS‐SSS was considered a clinically significant improvement [30].

#### 2.4.2 Secondary Outcomes

Secondary outcomes included assessments of psychosocial well‐being (quality of life (QoL), perceived stress, risk of depression and anxiety, fatigue, and pain catastrophizing experiences). These were also assessed at baseline, week 6, and week 12. QoL was assessed via the 34‐item IBS‐QoL questionnaire [31]. The 10‐item Perceived Stress Scale (PSS‐10) [32] was used to assess perceived stress. The Generalized Anxiety Disorder 7‐item scale (GAD‐7) [33] was used to determine the risk of anxiety. The 9‐item Patient Health Questionnaire (PHQ‐9) [34] was used to assess the risk of depression. Fatigue was measured using the 40‐item Functional Assessment of Chronic Illness Therapy (FACIT‐F) [35]. The pain catastrophizing experience was assessed using the 13‐item Pain Catastrophizing Scale (PCS).

##### 2.4.2.1 Assessment of Behaviors (Sleep and Physical Activity)

PA was measured using the 4‐item Godin Leisure Time Exercise questionnaire [36] and objectively (weekly steps, resting heart rate, and heart rate variability) using the Fitbit Inspire 3 worn for 12 weeks (starting at baseline). The Pittsburgh sleep quality index, a validated 10‐item sleep score (PSQI) [37], and Fitbit Inspire 3 were used to assess sleep scores. The Fitbit sleep score ranges from 1 to 100, with higher scores indicating better sleep based on the sum of sleep duration, deep and rapid eye movement sleep, and restoration [38].

##### 2.4.2.2 Feasibility

The recruitment rate was calculated as the percentage of eligible individuals who began the intervention out of all the eligible individuals approached to participate in the study. The attrition rate was calculated as the percentage of individuals dropping out of the intervention prior to its completion. For the LyfeMD app usage, metrics included the number of log‐ins, the total time spent using the app, and the engagement across app domains (Goal Tracking, Behavior Change Tools, PA, Diet, and YBM). Regarding the LyfeMD modules, the number of completed modules and the time required to complete all eight modules were recorded.

The questionnaire completion rate was determined by dividing the number of valid responses by the total possible responses completed across all questionnaires from individuals who completed the intervention. For valid data to be obtained from the Fitbit Inspire 3, the participant had to wear the tracker for at least 10 h per day [39, 40] with data for 1 week for at least four valid days [41]. To assess feasibility, the average number of valid days was calculated, along with the percentage of participants who met the threshold of recording sufficient data ≥ 4 valid days/week.

### 2.5 Statistical Methods

All data were analyzed using SPSS Statistics (v26, IBM), using intent‐to‐treat analysis. Continuous data were assessed for normality. Parametric data are presented with means and standard deviations, non‐parametric data with medians and confidence intervals, and categorical data with frequencies and percentages. Parametric and non‐parametric data were analyzed pre‐post with dependent *t*‐tests and Wilcoxon signed rank tests, respectively. Between group exploratory analysis used independent *t*‐tests and Mann–Whitney *U* tests for parametric and non‐parametric data, respectively. Individuals who participated in the previous Ascend IBS studies (REB‐20‐0084) and indicated a willingness to be contacted for future studies were invited to participate. These participants (*n* = 78) reported a mean score of 236 (SD + 85) on the IBS‐Symptom Severity Scale. Based on this estimate, to detect a clinically meaningful change of 50 points on the IBS‐Symptom Severity Scale (90% power and an alpha of 0.05), led to a minimum sample size of 30 participants. Assuming a conservative 20% loss to follow‐up, we enrolled a minimum of 36 participants in the study.

## 3 Results

### 3.1 Demographics

As seen in Table 1, 39 participants completed the intervention. The majority of participants were female (83.8%) with a mean age of 46.0 ± 13.7 years and a mean BMI of 28.4 ± 7.7 kg/m^2^. The most common medications prescribed to participants were selective serotonin or serotonin‐norepinephrine reuptake inhibitors (SSRIs/SNRIs (10/39: 25.6%)). The most common supplements were vitamins (22/39: 56.4%), followed by minerals (12/39: 30.8%), omega‐3 (4/39: 10.3%), and probiotics (3/39: 7.7%). Of the 39 participants, 25 used the entire LyfeMD platform (LyfeMD app + LyfeMD modules), while 13 used the LyfeMD app alone, with six of these individuals not having the opportunity to use the LyfeMD modules since they were not finalized at their time of participation and the remaining seven participants declining the LyfeMD modules.

**TABLE 1:** Participant baseline demographics.

Participant characteristics	Intervention (*n* = 39)
	Mean ± SD/median (IQR)/*n* (%)
Sex (female %)	33 (84.6)
Age	45.3 ± 13.7
BMI (kg/m^2^)	28.4 ± 7.7
Medications	
SSRI/SNRIs	10 (22.6)
TCAs	3 (7.7)
Bupropion	2 (5.1)
Ativan	2 (5.1)
Supplements	
Vitamins	22 (56.4)
Minerals	12 (30.8)
Omega‐3	4 (10.3)
Probiotics	3 (7.7)
IBS symptom severity score	
Mild (75–175)	2 (5.1)
Moderate (175–300)	23 (59.0)
Severe (> 300)	14 (35.9)
QoL (IBS‐QoL)	61.0 (43.4–76.5)
Stress (PSS‐10)	20.0 (13.0–26.0)
Anxiety (GAD‐7)	5.0 (3.0–9.0)
Depression (PHQ‐9)	8.0 (5.0–12.0)
Sleep (PSQI)	9.0 (6.0–11.0)
Fatigue (FACIT‐F)	96.0 (68.0–113.0)
Pain catastrophizing score	10.0 (3.0–24.0)
Mediterranean diet score	3.5 (2.5–4.8)
Healthy eating index	57.0 (50.5–69.0)

*Note:* Definitions: Selective serotonin reuptake inhibitors (SSRIs), Serotonin‐norepinephrine reuptake inhibitors (SNRIs), Tricyclic anti‐depressants (TCAs). IBS Quality of Life (IBS‐QoL): (0–100): higher scores indicating better QoL. Perceived Stress Scale (PSS‐10) (0–40): 0–13 low stress, 14–26 moderate stress, 27–40 high stress. Generalized Anxiety Disorder (GAD‐7) (0–21): 0–4 minimal anxiety, 5–9 mild anxiety, 10–14 moderate anxiety, ≥ 15 severe anxiety. Patient Health Questionnaire (PHQ‐9) (0–27): 1–4 minimal depression, 5–9 Mild, 10–14 moderate, 15–19 moderate–severe, 20–27 severe. Pittsburgh Sleep Quality Index (PSQI) (0–21): Higher scores indicate worse sleep quality, > 5 significant sleep difficulty. Functional Assessment of Chronic Illness Therapy‐Fatigue (FACIT‐F) (0–160): The higher the score the less fatigued. Pain Catastrophizing Score (0–52): Higher scores indicate higher level of catastrophizing; > 30 clinically significant.

### 3.2 IBS Symptoms

At the baseline assessment, the majority of participants (59.0%) reported moderate IBS symptom severity (IBSSS = 175–300). IBS symptom severity improved significantly (*Z* = −4.16; *p* < 0.001) from baseline (234.0 (182.0–320.0)) to 12 weeks (145.0 (104.8–260.0)), with initial changes observed as early as 6 weeks (196.5 (115.8–294.3)), with continued improvement over the final 6 weeks (*Z* = −1.92; *p* < 0.055). As a result, 63.2% of individuals reported a clinically significant improvement in their symptoms (≥ 50 points) over the 12 weeks. There were no differences in baseline age, sex, disease severity, or any of the psychosocial measures between the individuals who showed clinically significant improvement compared to those who did not. IBS symptom severity improvement was significantly associated with improvements in anxiety (rho = 0.477, *p* = 0.002), depression (rho = 0.474, *p* = 0.003), fatigue (rho = −0.625, *p* < 0.001), pain catastrophizing (rho = 0.606, *p* < 0.001), perceived stress (rho = 0.460, *p* = 0.004), and QoL (rho = −0.691, *p* < 0.001) (Table 2).

**TABLE 2:** Companson of patient‐reported outcomes from baseline to 6 and 12 weeks via the Wilcoxon signed‐rank test.

Statistic	Baseline score	6 week score			12 week score		
	Median (IQR)	Median (IQR)	*Z*	*p*	Median (IQR)	*Z*	*p*
IBS severity (IBSSS)	231.0 (180.5–312.0)	187.5 (105.3–246.8)	−3.60	< 0.001*	138.0 (104.3–256.8)	−4.11	< 0.001*
Stress (PSS‐10)	18.0 (13.0–26.0)	18.5 (13.25–22.0)	−1.27	0.205	16.0 (12.3–20.0)	−2.82	0.005*
Anxiety (GAD‐7)	5.0 (3.0–9.0)	5.0 (2.0–7.0)	−2.80	0.005*	2.5 (1.0–5.0)	−4.01	< 0.001*
Depression (PHQ‐9)	8.0 (5.0–12.0)	6.0 (3.0–11.0)	−2.41	0.016*	3.0 (2.0–8.8)	−4.34	< 0.001*
Sleep (PSQI)	9.0 (6.0–11.0)	7.0 (5.0–12.0)	−1.05	0.296	7.0 (4.3–9.8)	−2.76	0.006*
Fatigue (FACIT‐F)	98.0 (69.5–117.0)	106.0 (91.0–128.0)	−3.51	< 0.001*	112.0 (99.5–127.3)	−3.15	0.002*
Pain catastrophizing score	8.0 (3.0–23.5)	7.0 (1.0–15.0)	−2.87	0.004*	3.0 (1.0–11.5)	−2.72	0.006*

* Statistically significant: *p* < 0.05.

### 3.3 Psychosocial Outcomes

**FIGURE 1:**
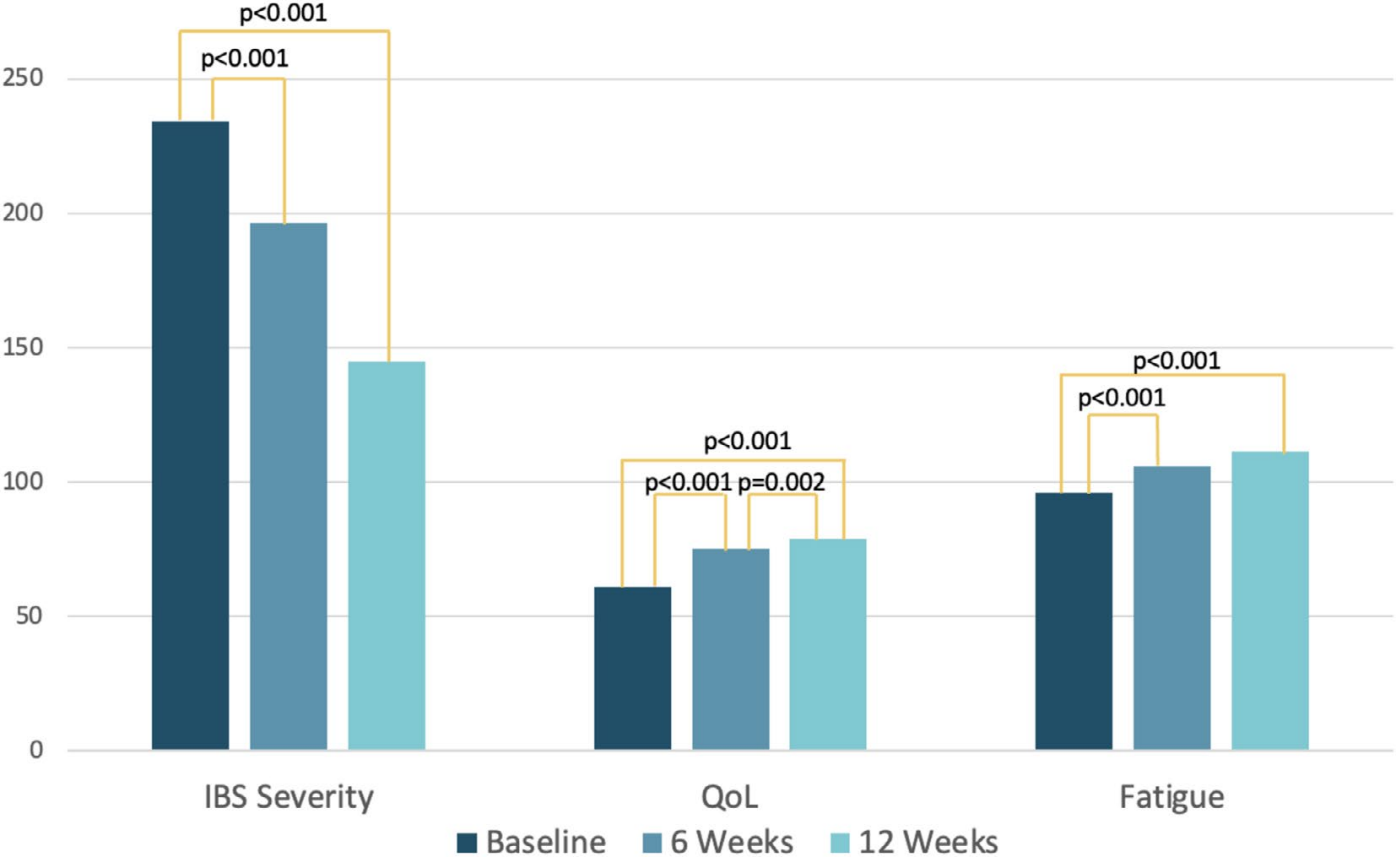
Self‐reported symptom severity, QoL, and fatigue across all time points. *High scores in QoL and fatigue indicate improvement in these symptoms and statistically significant: *p* < 0.05.

**FIGURE 2:**
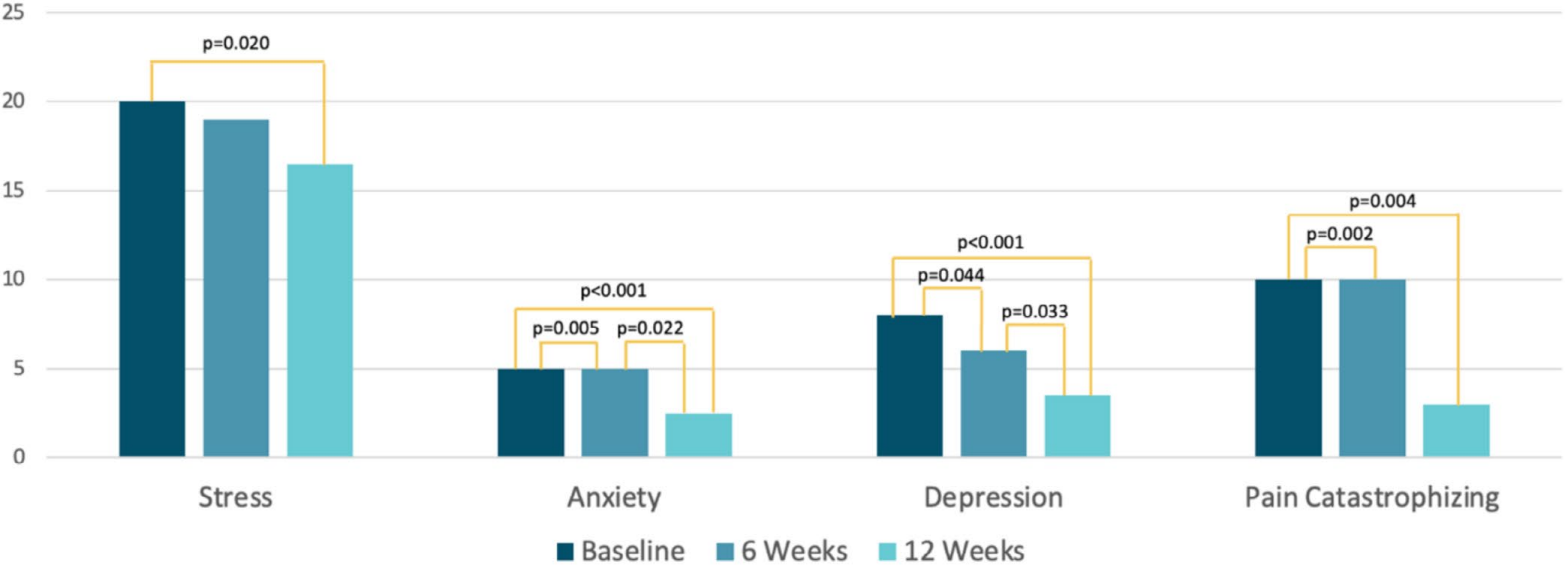
Self‐reported stress, anxiety, depression, and pain catastrophizing across all time points.

QoL (*Z* = −4.00; *p* < 0.001), anxiety (*Z* = −4.07; *p* < 0.001), depression (*Z* = −4.07; *p* < 0.001), fatigue (*Z* = −3.30; *p* < 0.001), pain catastrophizing (*Z* = −2.91; *p* < 0.004), and stress (*Z* = −2.33; *p* < 0.001) all improved significantly from baseline to 12 weeks (see Figures 1 and 2). While QoL, anxiety, and depression showed significant changes as early as 6 weeks of receiving the LyfeMD intervention, they showed further significant improvement in the last 6 weeks. Regarding QoL, 55.6% of participants showed a minimal important response to the intervention (≥ 10.2 points change [42]) and 47.2% a clinically meaningful difference (≥ 14 points change [42]). Individuals showing a clinically meaningful improvement had a significant (*t* = 2.67, *p* = 0.012) higher baseline BMI (BMI = 31.7 ± 8.7) compared to those that did not clinically improve (BMI = 25.4 ± 5.2). Fatigue significantly improved over the first 6 weeks and remained stable in the final 6 weeks. Perceived stress improved significantly over the 12 weeks, but not at 6 weeks. Individuals that completed 50% or more of the LyfeMD modules (*n* = 21/39) had significantly higher improvements in pain catastrophizing (∆PCS = −6.0 (−15.0 to −1.0); *Z* = −2.67; *p* = 0.007) and QoL (∆QoL = 16.9 (2.2–30.9); *Z* = −2.28; *p* = 0.023) compared to individuals that did not complete any LyfeMD modules (∆PCS = −1.0 (−4.0–4.0) and ∆QoL = 2.2 (−1.5–16.9)).

### 3.4 Sleep and Physical Activity

The majority of participants (87.2%) reported significant sleep difficulty (> 5 points on PSQI) at the beginning of the intervention, with a significant improvement over the 12 weeks of intervention (*Z* = −2.81; *p* = 0.005), resulting in 71.1% of participants still reporting significant sleep difficulty at the end of the intervention. For the individuals that wore the Fitbit, baseline quality of sleep was assessed to be fair (60–79 points on Sleep score) for 69.6% of participants and good (80–89 points on Sleep score) for the remaining 30.4% of participants. These scores did not significantly change over the 12‐week intervention.

At the beginning of the intervention, 51.3% of the participants met the general Canadian PA guidelines of more than 150 min of moderate to vigorous PA, with the average daily steps throughout the intervention being 7762 ± 3113 as recorded by the Fitbit Inspire 3. The individuals that met PA guidelines had a significantly (*Z* = −2.16, *p* = 0.031) lower stress score (14.5 (10.8–21.5)) compared to those that did not (23.0 (16.0–26.0)) at baseline. There was no significant association between PA levels and IBS symptom severity at baseline. Throughout the intervention, neither subjective PA levels nor steps measured by the activity tracker improved significantly.

### 3.5 Feasibility Data

**FIGURE 3:**
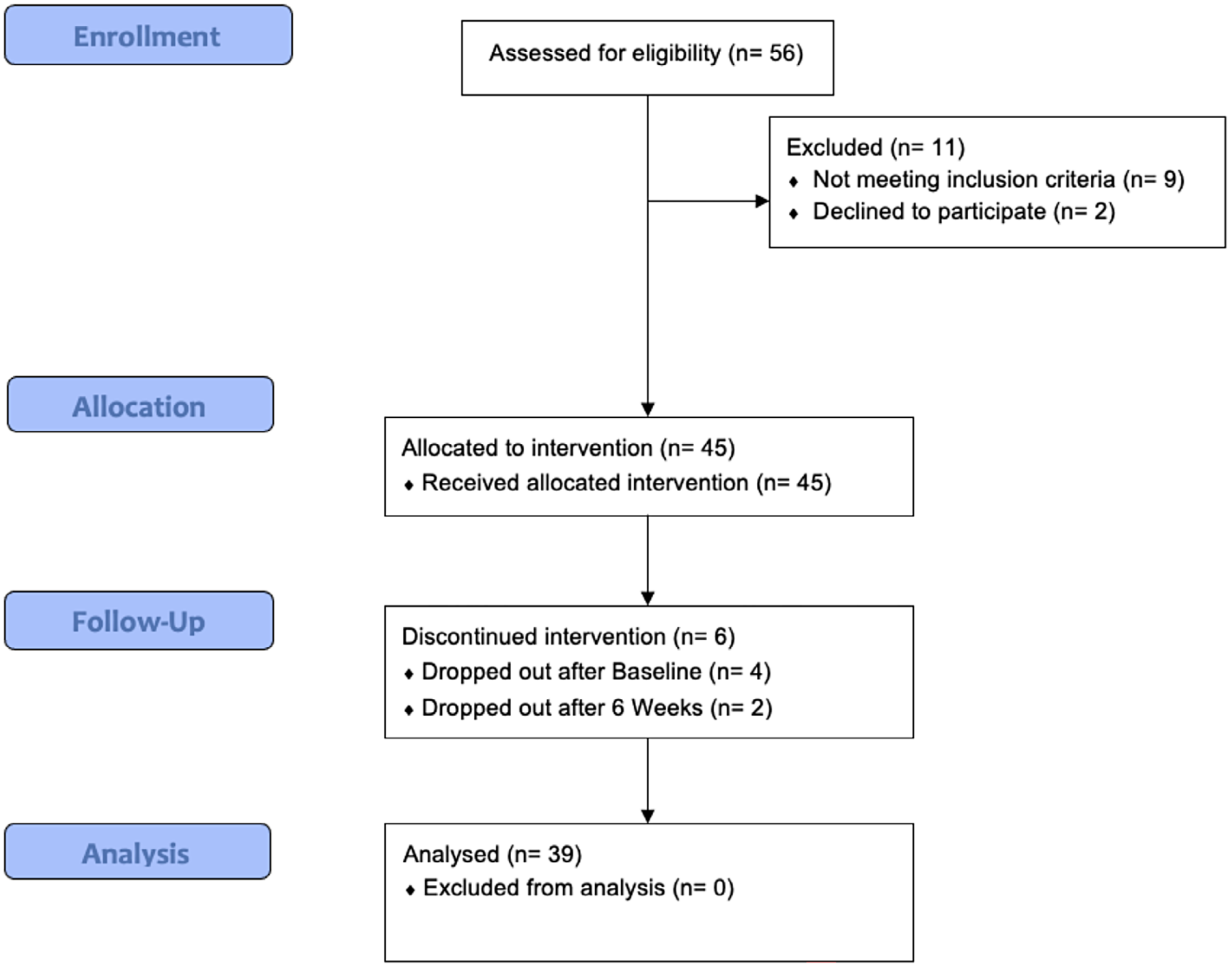
Consort flow diagram of participant recruitment, allocation, and attrition.

The overall recruitment rate was 95.8% (45/47) with two eligible participants declining study participation (Figure 3). The overall attrition rate was 13.3% (39/45), with four participants dropping out after the baseline assessment and two participants after the 6‐week assessment.

#### 3.5.1 Intervention Feasibility

The median time spent on the app per week was 2.9 (1.3–4.6) min, with one user utilizing the app as little as 0.2 min while another used it 16.5 min per week. There was no correlation between app usage and participant age, nor was an increased time spent on the application associated with improvements in the primary outcomes. The tools most often used in the application were the “Diet” and “YBM” domains, with 94.9% of participants (37/39) exploring both domains and a median session rate over the 12 weeks of 11.0 (2.0–30.0) and 14.0 (4.0–33.0) (Table S2). Of the 25 individuals that received access to the LyfeMD modules, 11 completed all 8 modules over a median duration of 36.0 (27.0–43.0) days, and 21 completed at least half of the modules. Four participants never completed any module, and the median number of completed modules out of eight was 7.0 (4.0–8.0). There was no correlation between the individuals that had high engagement in the LyfeMD applications and those that had high engagement in the LyfeMD modules (rho = −0.122, *p* = 0.560).

The HC was utilized by all participants and included a 40–45 min introductory call with three to four 30–35 min follow‐up calls. The behavior change tools utilized during the HC calls are outlined in Table S1.

#### 3.5.2 Assessment Feasibility

Participants wore the Fitbit tracker sufficiently on average 4.6 days per week throughout the intervention. 64.9% of the participants wore the tracker sufficiently (≥ 4 valid days/week) throughout all 12 weeks. The baseline, 6 weeks, and 12 weeks questionnaire completion rates were 99.6%, 95.4%, and 97.4% respectively.

## 4 Discussion

Dietary and behavioral modifications are key pillars in the management of IBS [8]. This pre‐post interventional trial aimed to assess the effectiveness of using the LyfeMD application in combination with HC to improve IBS symptoms and psychosocial wellbeing by facilitating mental health and lifestyle changes, including diet, yoga, breathing, mindfulness, sleep, and PA. Additionally, this study explored the feasibility of the intervention and the assessments.

In this study, IBS symptom severity and all psychosocial metrics—including QoL, stress, anxiety, depression, fatigue, and pain catastrophizing—improved significantly throughout the intervention. Notably, the reduction in symptom severity correlated with improvements in all psychosocial domains. While the study design precludes establishing causality or directionality, the observed relationship between concurrent improvements in IBS symptoms and psychosocial factors highlights the potential role of the gut‐brain axis in IBS pathophysiology [2]. A recent systematic review by D'Silva et al. on mHealth in IBS found similar findings, with 10 of the included 13 studies showing improvements in symptom burden. Additionally, in four out of the six studies that had a control group, the intervention group was superior to the control [43]. Finally, this review concluded that interventions more focused on gut‐brain behavior skills rather than diet alone appeared to have greater effectiveness, emphasizing the importance of focusing on interventions that incorporate psychosocial well‐being as one of the treatment modalities for IBS.

Interestingly, initial improvements in IBS symptom severity, QoL, anxiety, depression, and fatigue were observed as early as 6 weeks. Szigethy et al.'s single‐arm interventional study reported similar trends with improvement in mood symptoms as early as one month post‐initiation of an app‐based HC intervention [44]. Adopting mHealth solutions may lead to early clinical benefits, thereby contributing to sustainable health care systems [25]. In another randomized control trial by Lackner et al., minimal contact CBT was found to be non‐inferior in terms of symptom improvement compared to standard CBT (in‐person sessions) [45]. Therefore, mHealth interventions requiring a short duration of use may be attractive solutions from accessibility and cost perspectives; however, the durability of such improvements requires further study. Although none of the outcomes worsened in the latter 6 weeks, only QoL, anxiety, and depression showed further improvement. Unfortunately, in this pilot study, we did not have a follow‐up period to assess whether participants were able to maintain their improvements once the intervention was completed. Thus, a future RCT should investigate the minimal support an individual requires to accomplish the desired behavioral changes, with longer follow‐up periods to assess the maintenance phase of behavior change, including a cost analysis.

While the measured clinical outcomes improved with the intervention, the behaviors leading to these improvements (sleep and PA) are not readily apparent. One explanation for this may be that mindfulness—a behavior not measured in this study yet taught by HC and YBM modules, was the most utilized tool in the application which may have facilitated the observed improvements in symptom burden and psychosocial wellbeing. If mindfulness was a facilitating factor for symptom improvement this might highlight the importance of utilizing ACT based interventions in IBS, as a recent feasibility mHealth trial suggested [46]. Additionally, the skills learned through the HC through CBT have been shown previously to independently improve IBS symptoms [47]. The lack of improvement in the PA levels may be explained by the high baseline activity level of our sample compared to the usual low levels of PA in the IBS population [7]. Subjective sleep quality improved but objective sleep metrics did not. This discrepancy may be explained by the lack of validation of commercially available trackers to estimate sleep quality [48], the lack of specificity in sleep recognition of Fitbit devices in particular which is associated with an overestimation of sleep efficiency [49], as well as the prior established discrepancy between perceived and objective sleep quality [50]. A recent review of IBS patients found the same discrepancy and recommended placing greater emphasize on subjective sleep data. Additionally, the majority of studies in this review reported sleep disturbances to be associated with worsening IBS symptoms, highlighting the importance of addressing sleep as one of the key components of IBS management [51]. Diet was the second most utilized domain in the LyfeMD application with the “Eating plan” tool being utilized by most individuals. The intervention provided dietary advice based on the NICE guidelines and the low FODMAPs dietary recommendations. However, adherence to these recommendations was not assessed. One important consideration is that neither of these IBS‐specific dietary strategies directly target diet quality and may, in some cases, negatively impact it [52]. Therefore, future trials should consider assessing diet quality and adherence to IBS dietary therapies for example, by incorporating tools like the Comprehensive Nutrition Assessment Questionnaire (CNAQ) [53]—to gain a more nuanced understanding of participants' dietary changes.

In addition to the preliminary findings, this pilot study demonstrated the feasibility of conducting a fully powered RCT. Both attrition and recruitment rates were compared to similar remote trials [44]. However, large inter‐participant variability in LyfeMD app engagement may suggest differing levels of support required among patients with IBS. Although there were no associations between app usage and outcomes, participants who completed at least half of the LyfeMD modules had significantly higher improvements in QoL and pain catastrophizing. Additionally, the absence of an association between individuals' engagement in the LyfeMD app and in the LyfeMD modules may suggest that there were no “super users” but possibly that individuals personalized the intervention by choosing which of the behavior change tools (LyfeMD application, LyfeMD modules, or HC) fitted best to their needs. This hypothesis will be further examined by the planned follow‐up qualitative study by gaining a better understanding of the nuances in individual intervention utilization. The efficacy for enabling personalization of interventions to facilitate behavior change is still in its infancy, but preliminary findings are promising for its role in mHealth [54]. Finally, while the questionnaire completion rate was excellent, the Fitbit wear time should be improved in future trials for more reliable and complete objective data.

The limitations of this pilot study are the small sample size, the lack of a control group, and randomization procedures. Secondly, due to convenience sampling, our sample may have been biased by individuals that were already inclined to participate in lifestyle changes, which is supported by higher than expected baseline PA levels. Furthermore, although IBS is more prevalent in females, our representation of females was larger than expected of the IBS population and may therefore limit generalizability. Finally, the lack of a post‐intervention follow‐up period limits us from commenting on the sustainability of the observed changes. In a future fully powered RCT, consideration should be given to the aforementioned cost–benefit analysis, tracking of the content and duration of each HC call, improved usage data from the LyfeMD platform, and a post‐intervention follow‐up period of at least 3 months to assess sustainability of the behavior change. Additionally, more robust wearables to assess autonomic nervous system activity via heart rate variability [16], functional magnetic resonance imaging to assess adaptations in central connectivity, as well as stool and blood samples for microbiome assessment could be considered to help better understand the underlying biological mechanisms.

Overall, this pilot study provides compelling preliminary evidence that a digitally delivered, integrative lifestyle intervention, combining the LyfeMD mobile platform with personalized HC, can meaningfully improve symptom severity and psychosocial well‐being. The intervention was feasible and led to improvements in key clinical outcomes, including QoL, mental health, fatigue, and stress. Importantly, improvements in IBS symptoms correlated with psychosocial benefits, reinforcing the central role of the gut‐brain axis in IBS management. Despite modest engagement with some behavioral components such as PA and sleep, participants who engaged more with the modules experienced greater improvements, highlighting the value of structured digital health content. These findings highlight the potential of mHealth interventions not only to expand access to care, but to serve as effective complementary treatment modalities. Important lessons were learned to improve both the effectiveness and feasibility of a future fully powered randomized controlled trial.

## Author Contributions

Max Eisele: formal analysis, data curation, writing – original draft, visualization. Munazza Yousuf: investigation, data curation, writing – original draft. Natasha Haskey: methodology, writing – review and editing, supervision. Adrijana D'Silva: conceptualization, methodology, review and editing. Yasmin Nasser: conceptualization, methodology, review and editing. Laura Franco: investigation, data curation. Maitreyi Raman: conceptualization, methodology, validation, resources, data curation, writing – review and editing, supervision.

## Conflicts of Interest

Dr. Raman is the co‐founder and chief medical officer of LyfeMD. Dr. Haskey is a consultant for LyfeMD.

## Supporting information


**Data S1:** nmo70179‐sup‐0001‐Supplementaryfile1.docx.

**Data S2:** nmo70179‐sup‐0002‐Supplementaryfile2.doc.

**Data S3:** nmo70179‐sup‐0003‐Supplementaryfile3.pdf.

## Data Availability

The data that support the findings of this study are available on request from the corresponding author. The data are not publicly available due to privacy or ethical restrictions.
