# Supplementary material for: Smartphone Application With Health Coaching Facilitates Multi‐Symptom Improvement in IBS Patients: A Pilot Feasibility Trial
Source: Neurogastroenterol Motil. 2025 Oct 7;37(12):e70179. doi: 10.1111/nmo.70179 (PMC12623270; doi:10.1111/nmo.70179)
Supplement: Supplementary file 1 — Data S1: nmo70179‐sup‐0001‐Supplementaryfile1.docx. [file NMO-37-e70179-s001.docx]

**SUPPLEMENTARY FILE 1.**

Table 1.Behaviour Change Tools utilized in the LyfeMD application and during Health Coaching calls based on the Behaviour Change Taxonomy^1^

|  | Health Coaching | LyfeMD application |
| --- | --- | --- |
| BCT domain | BCTs | |
| 1. Goal setting and planning | 1.1 Goal setting (behaviour) | 1.1 Goal setting (behaviour) |
|  | 1.3 Goal setting (outcome) | 1.3 Goal setting (outcome) |
|  | 1.4 Action planning | 1.4 Action planning |
|  | 1.7 Review outcome goals | 1.7 Review outcome goals |
| 2. Feedback and monitoring | 2.1 Monitoring of behaviour by others without feedback | 2.1 Monitoring of behaviour by others without feedback |
|  | 2.3 Self-Monitoring of behaviour | 2.3 Self-Monitoring of behaviour |
|  | 2.4 Self-monitoring of outcome of behaviour | 2.4 Self-monitoring of outcome of behaviour |
|  | 2.7 Feedback on outcome of behaviour |  |
| 3. Social Support | 3.1 Social support (unspecified) |  |
|  | 3.2 Social support (practical) |  |
|  | 3.3 Social support (emotional) |  |
| 4. Shaping knowledge | 4.1 Instruction on how to perform a behaviour | 4.1 Instruction on how to perform a behaviour |
| 5. Natural consequences | 5.1 Information about health consequences | 5.1 Information about health consequences |
|  |  | 5.4 Monitoring of emotional consequences |
|  |  | 5.6 Information about emotional consequences |
| 6. Comparison of behaviour |  | 6.1 Demonstration of the behaviour |
| 7. Associations | 7.1 Prompts/cues | 7.1 Prompts/cues |
| 8. Repetition and substitution | 8.2 Behavior substitution |  |
|  | 8.7 Graded tasks | 8.7 Graded tasks |
| 9. Comparison of outcomes | 9.1 Credible source | 9.1 Credible source |
| 11. Regulation | 11.2 Reduce Negative Emotions |  |
| 12. Antecedents |  | 12.5 Adding objects to the environment |
| 13. Identity | 13.2 Framing/ reframing |  |
| 15. Self-belief | 15.1 Verbal persuasion about capability |  |
|  | 15.3 Focus on past successes |  |

1. Michie S, Richardson M, Johnston M, et al. The behavior change technique taxonomy (v1) of 93 hierarchically clustered techniques: building an international consensus for the reporting of behavior change interventions. *Annals of behavioral medicine*. 2013;46(1):81-95.

Table 2. Median LyfeMD application utilization per participant over the 12 week course of the intervention

|  | Median LyfeMD sessions (IQR) |
| --- | --- |
| Total | 19 (9 – 31) |
| Diet | 11 (2 – 30) |
| Yoga Breathing Mindfulness | 14 (4 – 33) |
| Behavior Change tools | 2 (0 – 4) |
